# Evaluation of ‘implications for research’ statements in systematic reviews of interventions in advanced cancer patients – a meta-research study

**DOI:** 10.1186/s12874-023-02124-y

**Published:** 2023-12-20

**Authors:** W. Siemens, G. Bantle, C. Ebner, A. Blümle, G. Becker, G. Schwarzer, J. J. Meerpohl

**Affiliations:** 1 Institute for Evidence in Medicine, Faculty of Medicine, Medical Center, University of Freiburg, University of Freiburg, Freiburg, Germany Breisacher Str. 86, 79110; 2 Cochrane Germany, Cochrane Germany Foundation, Freiburg, Germany; 3 Clinical Trials Unit, Faculty of Medicine and Medical Center, University of Freiburg, Freiburg, Germany; 4 Department of Palliative Medicine, Faculty of Medicine, University Medical Center Freiburg, University of Freiburg, Freiburg, Germany; 5 Institute of Medical Biometry and Statistics, Faculty of Medicine and Medical Center, University of Freiburg, Freiburg, Germany

**Keywords:** Systematic reviews, Implications for research, Meta-research, GRADE, Oncology

## Abstract

**Background:**

Implications for research (IfR) sections are an important part of systematic reviews (SRs) to inform health care researchers and policy makers. PRISMA 2020 recommends reporting IfR, while Cochrane Reviews require a separate chapter on IfR. However, it is unclear to what extent SRs discuss IfR.

We aimed i) to assess whether SRs include an IfR statement and ii) to evaluate which elements informed IfR statements.

**Methods:**

We conducted a meta-research study based on SRs of interventions in advanced cancer patients from a previous project (CRD42019134904).

As suggested in the Cochrane Handbook, we assessed if the following predefined variables were referred to in IfR statements: patient, intervention, control, outcome (PICO) and study design; concepts underlying Grading of Recommendations, Assessment, Development and Evaluation (GRADE) domains: risk of bias, inconsistency, indirectness, imprecision, publication bias. Data were independently extracted by three reviewers after piloting the data extraction form. Discrepancies were resolved in weekly in-depth discussions.

**Results:**

We included 261 SRs. The majority evaluated a pharmacological intervention (*n* = 244, 93.5%); twenty-nine were Cochrane Reviews (11.1%).

Four out of five SRs included an IfR statement (*n* = 210, 80.5%). IfR statements commonly addressed ‘intervention’ (*n* = 121, 57.6%), ‘patient ‘ (*n* = 113, 53.8%), and ‘study design’ (*n* = 107, 51.0%). The most frequent PICO and study design combinations were ‘patient and intervention ‘ (*n* = 71, 33.8%) and ‘patient, intervention and study design ‘ (*n* = 34, 16.2%).

Concepts underlying GRADE domains were rarely used for informing IfR recommendations: ‘risk of bias ‘ (*n* = 2, 1.0%), and ‘imprecision ‘ (*n* = 1, 0.5%), ‘inconsistency ‘ (*n* = 1, 0.5%).

Additional elements informing IfR were considerations on cost effectiveness (*n* = 9, 4.3%), reporting standards (*n* = 4, 1.9%), and individual patient data meta-analysis (*n* = 4, 1.9%).

**Conclusion:**

Although about 80% of SRs included an IfR statement, the reporting of PICO elements varied across SRs. Concepts underlying GRADE domains were rarely used to derive IfR. Further work needs to assess the generalizability beyond SRs in advanced cancer patients. We suggest that more specific guidance on which and how IfR elements to report in SRs of interventions needs to be developed. Utilizing PICO elements and concepts underlying GRADE according to the Cochrane Handbook to state IfR seems to be a reasonable approach in the interim.

**Registration:**

CRD42019134904.

**Supplementary Information:**

The online version contains supplementary material available at 10.1186/s12874-023-02124-y.

## Background

Systematic reviews are commonly used to summarize the evidence from primary studies regarding a specific clinical question. Implications for research (IfR) are an important part of systematic reviews in healthcare and may facilitate an efficient interaction between players involved in evidence synthesis, primary research, guideline development, health technology assessment, and health policy [1]. IfR, derived from the main results of a systematic review, can be stated *after* new findings of clinical trials are integrated in the body of evidence of the corresponding systematic review [2]. Conversely, systematic reviews and its IfR are also important *before* conducting a new study as elaborated by the EVBRES (EVidence-Based RESearch) network in the ‘evidence-based research approach’ [2, 3].

The Lancet Series *Research: increasing value, reducing waste* already criticized in 2014 that new primary research is often conducted without justification by a systematic review, which could result in redundant or misleading research at the expense of patients’ health, quality of life, and in wasted resources [4, 5]. However, systematic reviews to inform the conduct of new studies are still carried out in only 16% to 87% [6] or 0% to 73% [7] to justify new studies.

The Preferred Reporting Items for Systematic reviews and Meta-Analyses (PRISMA) statement states in Item 23d: ‘Discuss implications of the results for practice, policy, and future research’ [8]. Additionally, the Cochrane Handbook defines an obligatory IfR chapter as second subchapter for the authors’ conclusions in Cochrane Reviews [9]. The Cochrane Handbook [9] and other sources [3, 10] suggest considering PICO elements (i.e., patient, intervention, control, and outcome) in IfR sections. Referring to PICO in the context of the certainty of evidence according to the Grading of Recommendations, Assessment, Development and Evaluation (GRADE) approach when stating IfR is considered ‘helpful’ in the Cochrane Handbook [9]. Importantly, IfR referring to GRADE are specific to certain outcomes and can be derived from each GRADE domain, e.g., pointing out the need for an individual participant data meta-analysis or subgroup analysis in the case of unexplained inconsistency [9].

While we know that systematic reviews could be used more often to justify future studies [2, 4, 6, 7], it remains unclear how many systematic reviews actually contain an IfR statement and if these statements are structured in an informative and useful way to help conduct future studies.

For this purpose, we first aimed to assess whether systematic reviews included an IfR statement. For those systematic reviews *with* an IfR statement, our second aim was to analyze whether the IfR statement considered PICO elements, concepts underlying GRADE domains, or additional IfR elements.

## Methods

### Study design

We conducted a meta-research study to answer the study aims. Our sample were systematic reviews of interventions in patients with advanced cancer from a previous project (CRD42019134904) [11, 12]. Results are reported in line with the PRISMA guideline as far as applicable for our study design [8].

### Eligibility criteria

We used systematic reviews of interventions as unit of analysis with at least one statistically significant meta-analysis of at least four randomized controlled trials (RCTs) per review (see [11, 12] for further details). We included systematic reviews with pharmacological, surgical, and radiotherapeutic interventions for advanced cancer patients. There were no limitations regarding the control group.

We excluded systematic reviews with the following characteristics: reviews assessing non-randomized studies of interventions, network meta-analyses, prognostic reviews, reviews on validation or diagnosis, scoping reviews, and outdated Cochrane Reviews.

### Search

Medline (via Ovid), the Cochrane Database of Systematic Reviews (via Wiley) and Web of Science (Science Citation Index Expanded) had been searched from January 2010 to July 2019 See search strategy and further details in [11, 12].

### Selection process and data extraction

For the previous project, two reviewers screened the search results independently, selected the relevant systematic reviews, and extracted data on review characteristics and methodological quality according to A Measurement Tool to Assess Systematic Reviews (AMSTAR) 2 [11–13].

In the present meta-research study, three reviewers (WS, GBa, CE) extracted predefined IfR variables. We used the Cochrane Handbook (Chapter 15.6.3) to define and structure our IfR variables [14]. We assessed whether IfR reported PICO (i.e., patient, intervention, control, outcome), study design and whether concepts underlying GRADE domains (risk for bias, inconsistency, indirectness, imprecision, publication bias) [15] were used in IfR statements to describe shortcomings in the body of evidence and to derive IfR. GRADE terminology or related expressions, e.g. “unprecise” or “wide confidence intervals” to address “imprecision”, had to be used by systematic review authors to rate methodological concepts underlying GRADE domains as ‘yes’ in data extraction. IfR statements related to ‘adequate sample size’ and ‘power’ of a future trial were extracted as additional IfR element because they are a possible consequence of an ‘imprecise’ result and not the methodological shortcoming itself, which is usually described by methodological concepts underlying GRADE. Such additional IfR elements were not predefined but similar IfR aspects were categorized and summarized inductively, e.g., terms like ‘CONSORT’ [Consolidated Standards of Reporting Trials], ‘standardization of outcome assessment’ and ‘correct reporting of outcomes’ were summarized with ‘reporting standards’; or ‘benefits balanced with costs’, ‘incremental cost’ and ‘resource use and cost effectiveness analyses’ were summarized with the term ‘cost effectiveness’.

Moreover, we extracted if an explicit stop statement for future research was stated by the review authors and we extracted a quote of the stop statement if applicable. We defined a stop statement *as an explicit statement that an additional trial to answer the review question overall or regarding a certain aspect (e.g., subgroup, endpoint) is no longer needed*. The extraction form and data are available at the Open Science Framework: https://osf.io/y9v4x/.

We piloted the extraction process using the first five systematic reviews, which were extracted and thoroughly discussed by three reviewers. In the subsequent data extraction process for the remaining systematic reviews, three reviewers (WS, GBa, CE) extracted the data separately and resolved arising discrepancies in weekly in-depth discussions.

### Defining an IfR statement

We defined an *IfR statement as at least one sentence, which contains at least one bit of information that could be informative for planning future research*, e.g. an element from the PICO scheme. Systematic reviews containing only uninformative IfR statements such as ‘more research is needed’ were not accepted and rated as ‘no IfR statement’ in data extraction.

### Sample

In a first step, we describe the total sample of systematic reviews and assess whether they included an IfR statement (aim 1).

In a second step, we examined a subsample of those systematic reviews *with* an IfR statement to assess whether PICO elements, study design, concepts underlying GRADE domains, or if additional elements were mentioned in IfR statements (aim 2).

### Statistical analysis

We used descriptive statistics with absolute and relative frequencies to summarize categorical outcomes. Means and standard deviations were used to describe results of continuous outcomes. We analyzed frequencies of PICO elements, study design and concepts underlying GRADE domains addressed in IfR and PICO/study design combinations (e.g., ‘patient and intervention‘). We used the statistical program R (version 4.1.2) [16].

## Results

### Characteristics of included systematic reviews

**Fig. 1:**
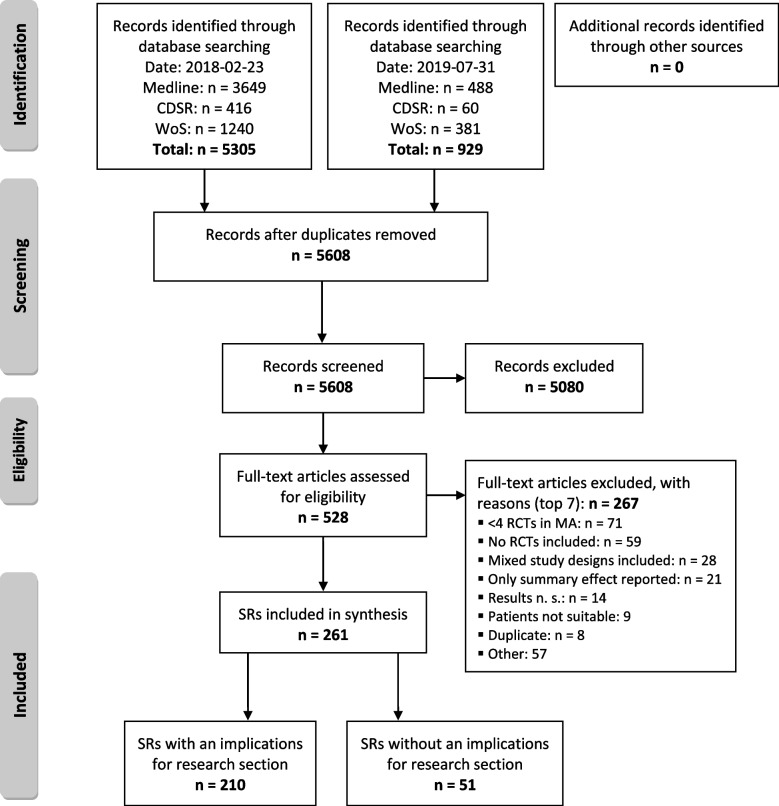
Flow Diagram of included systematic reviews; *CDSR* Cochrane Database of Systematic Reviews; *MA* meta-analysis; *n. s.* not statistically significant; *PRISMA* Preferred reporting items for systematic reviews and meta-analyses; *RCT* randomized controlled trial; *SR* Systematic Review; *WoS* Web of Science

In total, 261 systematic reviews were included in our data set, 210 (80.5%) of those *with* an IfR statement and 51 (19.5%) *without* (see Fig. 1).

The characteristics of the included systematic reviews are presented in Table 1. The majority of systematic reviews (26.1%) included patients with lung cancer and 93.5% evaluated a pharmacological treatment. In 59.8% the control group was standard care and only about in half (51.7%) of the systematic reviews a primary outcome was defined, with overall survival as the most frequent one (32.2%). GRADE was rarely used in these systematic reviews (11.5%). The majority of systematic reviews (88.9%) were not Cochrane Reviews, most of them with a critically low methodological quality according to AMSTAR 2 (88.1%). Systematic reviews *with* and *without* an IfR statement were comparable for most characteristics, e.g., patients, control, and primary outcome of review. We observed possible associations for systematic reviews *with* IfR, which addressed clinical relevance of results more likely, and for Cochrane Reviews (variable: review type). Cochrane Reviews have an obligatory IfR section, use GRADE and usually meet most criteria of AMSTAR 2, as possibly indicated by the differences of these variables for systematic reviews *with* and *without* an IfR statement (Table 1).

**Table 1:** Characteristics of included systematic reviews: total and IfR statement: yes/no

	**Total**	**IfR: Yes**	**IfR: No**
	***N* = 261 (%)**	***n* = 210 (%)**	***n* = 51 (%)**
Patients:			
Lung cancer	68 (26.1)	53 (25.2)	15 (29.4)
Colorectal cancer	45 (17.2)	35 (16.7)	10 (19.6)
Breast cancer	31 (11.9)	26 (12.4)	5 (9.8)
Gastric cancer	30 (11.5)	24 (11.4)	6 (11.8)
Pancreatic cancer	19 (7.3)	16 (7.6)	3 (5.9)
Mixed types of cancer	17 (6.5)	12 (5.7)	5 (9.8)
Genitourinary cancer	11 (4.2)	9 (4.3)	2 (3.9)
Brain metastases	10 (3.8)	9 (4.3)	1 (2.0)
Esophageal cancer	9 (3.4)	8 (3.8)	1 (2.0)
Hepatocellular carcinoma	6 (2.3)	5 (2.4)	1 (2.0)
Bone metastases	3 (1.1)	3 (1.4)	0 (0.0)
Other	12 (4.6)	10 (4.8)	2 (3.9)
Intervention:			
Pharmacological treatment	244 (93.5)	195 (92.9)	49 (96.1)
Radiotherapy	10 (3.8)	10 (4.8)	0 (0.0)
Surgery	7 (2.7)	5 (2.4)	2 (3.9)
Control:			
Standard care	156 (59.8)	131 (62.4)	25 (49.0)
Standard care and active comparator	57 (21.8)	42 (20.0)	15 (29.4)
Standard care and placebo	41 (15.7)	31 (14.8)	10 (19.6)
Same intervention but modified (e.g. different doses)	7 (2.7)	6 (2.9)	1 (2.0)
Primary outcome of review:			
Not defined	126 (48.3)	95 (45.2)	31 (60.8)
Overall survival	84 (32.2)	70 (33.3)	14 (27.5)
Progression-free survival	21 (8.0)	19 (9.0)	2 (3.9)
Complete response	12 (4.6)	10 (4.8)	2 (3.9)
Disease event	5 (1.9)	4 (1.9)	1 (2.0)
Other	13 (5.0)	12 (5.7)	1 (2.0)
Use of GRADE:			
No	231 (88.5)	183 (87.1)	48 (94.1)
Yes	30 (11.5)	27 (12.9)	3 (5.9)
Addressing clinical relevance of results:			
No	151 (57.9)	113 (53.8)	38 (74.5)
Yes	110 (42.1)	97 (46.2)	13 (25.5)
Type of review:			
Neither using PRISMA nor a Cochrane Review	129 (49.4)	100 (47.6)	29 (56.9)
Review using PRISMA	103 (39.5)	81 (38.6)	22 (43.1)
Cochrane Review	29 (11.1)	29 (13.8)	0 (0.0)
Registration of review:			
No	222 (85.1)	174 (82.9)	48 (94.1)
Yes	39 (14.9)	36 (17.1)	3 (5.9)
AMSTAR 2 overall rating:			
critically low	230 (88.1)	180 (85.7)	50 (98.0)
high	12 (4.6)	12 (5.7)	0 (0.0)
low	11 (4.2)	10 (4.8)	1 (2.0)
moderate	8 (3.1)	8 (3.8)	0 (0.0)

*AMSTAR* A MeaSurement Tool to Assess Systematic Reviews, *GRADE* Grading of Recommendations, Assessment, Development and Evaluation, *IfR* Implications for research, *PRISMA* Preferred Reporting Items for Systematic Reviews and Meta-Analyses, *SD* Standard deviation

### IfR results

For answering the second questions of this meta-research study, we focused on the systematic reviews *with* an IfR statement (*n* = 210) and examined if PICO elements, study design, concepts underlying GRADE domains, or additional elements were reported in IfR statements (Table 2).

**Table 2:** Results of systematic reviews with an IfR statement

IfR results	Total
	***N* = 210 (%)**
Location of IfR statement:	
Discussion	137 (65.2)
Conclusion	44 (21.0)
Both, discussion and conclusion	29 (13.8)
IfR statement and primary outcome:	
Primary outcome not defined	95 (45.2)
IfR do not refer to primary outcome	90 (42.9)
IfR do refer to primary outcome	25 (11.9)
PICO elements (alone or any combination):^a^	
Intervention	121 (57.6)
Patient	113 (53.8)
Study design	107 (51.0)
Outcome	55 (26.2)
Control	27 (12.9)
Relevant combinations of PICO elements and study design:^b^	
Patient and intervention	71 (33.8)
Patient, intervention and study design	34 (16.2)
Patient, intervention and outcome	21 (10.0)
Patient, intervention, outcome, study design	8 (3.8)
Concepts underlying GRADE domains addressed:^c^	
Risk for bias	2 (1.0)
Imprecision	1 (0.5)
Inconsistency	1 (0.5)
Not mentioned	206 (98.1)
Additional IfR elements:^d^	
Costs effectiveness	9 (4.3)
Reporting standards	4 (1.9)
IPD meta-analysis	4 (1.9)
Stop statement for further research:	
No	207 (98.6)
Yes	3 (1.4)

*IPD* Individual patient data, *PICO* patient intervention, control, and outcome

^a^ Numbers do not add up to 210 due to multiple counting

^b^ Numbers do not add up to 210 because the chosen combinations were of high interest and were selected from various other combinations

^c^ Combinations of GRADE domains not assessed due to their low frequency

^d^ Open category in data extraction, which allowed for no data and multiple additional IfR elements per systematic review

IfR statements were mostly placed in the discussion (*n* = 137, 65.2%) and referred to the primary outcome (*n* = 25, 11.9%) of the systematic reviews *with* an IfR-statement. Commonly addressed elements in IfR statements were ‘intervention ‘ (*n* = 121, 57.6%), ‘patient‘ (*n* = 113, 53.8%) and ‘study design’ (*n* = 107, 51.0%). As a sub-aspect of ‘patients’, ‘biomarkers’ were mentioned in the IfR statement of 21 (10.0%) systematic reviews. Combinations of IfR PICO elements and study design were stated in 71 (33.8%) systematic reviews for ‘patient and intervention’ and in 34 (16.2%) for ‘patient, intervention and study design’. All elements in combination except ‘control’ were reported in 8 (3.8%) systematic reviews. Other, infrequent combinations of IfR PICO elements and other elements are shown in Appendix 1.

Concepts underlying GRADE domains to describe the shortcomings of the body of evidence of an outcome were rarely used to derive IfR: ‘risk of bias’ (*n* = 2, 1.0%), ‘imprecision’ (*n* = 1, 0.5%), and ‘inconsistency’ (*n* = 1, 0.5%). In twenty-eight (13.3%) of the systematic reviews ‘appropriate sample size/power’ was addressed in the IfR statement.

Table 3 gives sample excerpts from multiple IfR statements identified in the discussion and conclusion from two Non-Cochrane Reviews (Lee et al., Wieser et al.) and a Cochrane Review IfR section (Pasquali et al.). Beside different PICO elements, individual patient data (IPD) or reporting standards were for example mentioned as additional IfR elements. Imprecision and risk of bias were stated as methodological concepts underlying GRADE in an IfR section (Pasquali et al.) or in context of an IfR statement (Lee et al.).

**Table 3:** Sample excerpts of IfR statements

IfR statements	IfR elements
Pasquali et al., 2018, https://doi.org/10.1002/14651858.CD011123.pub2:	
“Randomised controlled trials with longer **follow-up periods** (12 to 24 months) for participants treated with **new therapeutic agents immune checkpoint inhibitors and targeted therapies** are needed to assess impact on **overall survival**. Other outcomes that need to be assessed include **quality of life** and issues relating to health economics, such as **cost-effectiveness**.”	PICO elements: Patient, intervention, outcome, time frame
“Future published trials should guarantee adequate reporting by adhering to guidelines such as **CONSORT**.” “Identification of **biomarkers** for guide selection of people most responsive”	Additional IfR elements: cost-effectiveness, reporting standards (CONSORT), biomarker
“A common reason for downgrading evidence quality was **imprecision**“	GRADE concepts: Imprecision
Lee et al., 2017, https://doi.org/10.1159/000446115:	
“Further trials, particularly investigating the combination of **bevacizumab** with other **targeted therapies**, are warranted.”	PICO elements: Patient, Intervention, Study design
“In addition, the demonstration of a PFS benefit overall strongly argues for ongoing research into the best way to **sequence these agents** in the treatment paradigm for **NET [neuroendocrine tumors]**” “However, this will need to be assessed in an **RCT**.”	Additional IfR elements: individual patient data (IPD) meta-analysis
“The use of **individual patient data meta-analyses** would decrease **the risk of bias** and provide greater statistical certainty regarding the benefit of **specific targeted agents**, and it would allow further **subgroup analyses**.”	GRADE concepts: Risk of bias
Wieser et al., 2010; https://doi.org/10.1186/1471-2407-10-309:	
“Thus, further clarification of **which patient group** would benefit by **perioperative chemotherapy**, whether **applied pre- or post-operatively**, and **which drug or combination of drugs** would be most effectively applied, is essential”	PICO elements: Patient, intervention, outcome
“The results of further studies will hopefully elucidate the most suitable treatment modality in **operable patients**.” “… further efforts to **improve chemotherapeutic regimens** to **minimize toxicities** are clearly warranted.”	Additional IfR elements: IPD meta-analysis
“The results must therefore be interpreted cautiously, as an **IPD-based meta-analysis** would give a more reliable estimation than one based on abstracted data.” And: “Our results should be confirmed by an **IPD-based meta-analysis**.”	GRADE concepts: None

*GRADE* Grading of Recommendations Assessment, Development, and Evaluation, *PICO* patient, intervention, control, outcome, *RCT* randomized controlled trial

Frequent additional elements mentioned in the context of IfR were cost effectiveness (9, 4.3%), reporting standards (4, 1.9%), and individual patient data meta-analysis (4, 1.9%) (Table 2).

Stop statements were very rare and, if present (*n* = 3), authors stated that results are ‘unlikely to change’, ‘further research […] is likely not necessary’, or ‘meta-analysis suggests that we do not need another trial’ (see Table 4).

**Table 4:** Stop statements in three systematic reviews with an IfR statement

Review	Stop statement in IfR statement
Ghersi et al., 2015, https://doi.org/10.1002/14651858.CD003366.pub3	“Breast cancer management has evolved considerably since the first version of this review. Specifically, there is an increasing emphasis on the different biological subtypes of breast cancer and a rapidly developing array of targeted therapies to be used in place of or as adjuncts to cytotoxic chemotherapy. Thus the results of this review, which was confined to trials of chemotherapy alone, are unlikely to change, and further updates are not planned.”
Kunath et al., 2014, https://doi.org/10.1002/14651858.CD009266.pub2	“The quality of evidence according to GRADE is only moderate. However, we believe that further research on non-steroidal antiandrogen monotherapy is likely not necessary for the subgroup of men with metastatic prostate cancer.”
Non-Small Cell Lung Cancer Collaborative Group, 2010, https://doi.org/10.1002/14651858.CD007309.pub2	“The current meta-analysis suggests that we do not need another trial of supportive care alone versus supportive care and chemotherapy.”

*GRADE* Grading of Recommendations Assessment, Development, and Evaluation

## Discussion

This meta-research study showed that four out of five systematic reviews assessing treatments in advanced cancer patients had an IfR statement.

IfR statements were informed by ‘intervention’, ‘patient’ and ‘study design’, mentioned alone as well in combination, in > 50% of systematic reviews. Systematic review authors referred rarely to ‘control’ in IfR statements although IfR information on the control might be very helpful to design a meaningful trial. E.g., it makes a substantial difference for planning a trial and/or using trial results for guideline recommendations if an intervention would be compared against placebo or against the current gold standard. Further, the IfR statements focused only on few combinations of PICO elements when stating IfRs and were most often limited to ‘patients and interventions’. Concepts underlying GRADE domains were practically not mentioned by systematic review authors when stating IfR recommendations.

### IfR in context of the evidence-based research approach

Strong efforts have been made to emphasize the relevance of systematic reviews to reduce research waste [4, 5] by the EVBRES network, in which an evidence-based research approach was endorsed [2, 3]. Such research activities underline the relevance of the topic and, when going in more detail of the evidence-based research framework, also support the idea of precise and useful IfR sections. However, recent analyses on the use of systematic reviews to inform the conduct of new studies suggest that there is still a long way to go to make this a common standard [6, 7]. This is in line with the findings of this meta-research study with a closer look to the quality of the IfR statements. Although an IfR was stated in the majority of the systematic reviews, it is questionable if the conduct of a future study could have been informed adequately by most of these IfR statements.

In accordance with the EVBRES network [2], we support the idea that IfR should be put into context of a research process and can be understood as an important gear in the evidence ecosystem in health care. Moreover, considering IfR *before* and *after* the conduct of a new study fits well to the idea of considering not only PICO elements when stating IfR but also reflecting on the certainty of evidence of each outcome according to concepts underlying GRADE domains [9]. In the context of continuous emergence of new evidence [17], we suggest that IfR sections may use concepts underlying GRADE for assessing the body of evidence per outcome and also state IfR per outcome (O) using specifications for the remaining elements: patients, intervention, control, study design (PICS). Additional elements may be added to the IfR depending on the specific context of a medical research field. This approach is likely to be an iterative process until methodological and clinical criteria are met to stop future studies or declare conclusiveness of a research question.

### Stop criteria / conclusiveness in IfR sections

Deciding whether and how to define stop criteria for future studies is extremely complex but nevertheless very important to avoid unnecessary studies in the case of a very conclusive and robust findings. Stop statements for additional studies are per definition a form of IfR statements and should not be confused with stopping rules of interim analyses within clinical trials, e.g., stopping for futility [18]. Our findings suggest that reasons for stopping further studies were worded in a very generic way and may trigger questions on explicit criteria to justify such stop statements. Meta-research on 545 Cochrane Reviews, which were labeled as stable or closed, showed a variety of reasons for stabilizing a Cochrane Review. They reached from ‘Last search did not identify any potentially relevant studies likely to change the conclusions’ (99, 18.2%) and ‘Research area no longer active’ (86, 15.8%) to ‘A new search within 2 years is not likely to identify any potentially relevant studies likely to change the conclusions’ (22, 4.0%) [19]. ‘Evidence is conclusive’ was stated in (35, 6.4%) cases leading to a subsequent project analyzing this subsample in an updated analysis with 39 Cochrane Reviews, for which Cochrane declared evidence was conclusive or will likely not change with the inclusion of further studies. Categorizing the rationale for stabilization in definitive, non-definitive, and ambivalent wording revealed a similar pattern identified in our results, i.e., the statements were very generic without referring to clear criteria, pattern, or algorithm for defining the conclusiveness of the review [20].

### Limitations

This analysis of IfR statements refers to a sample from a previous project investigating the methodological quality and statistical heterogeneity of systematic reviews in the field of oncology (see [11, 12] for further details). The sample consists of systematic reviews with at least one statistically significant meta-analysis. Lung cancer patients and especially pharmacological interventions were very frequent. Therefore, the generalizability of IfR findings is limited and results cannot necessarily be applied to other medical fields. We expect that especially additional IfR elements (e.g., biomarker) could vary depending on the medical field. A similar IfR analysis for a sample of systematic reviews assessing treatments for COVID-19 [21] is underway and will add important information regarding consistency and generalizability of the findings in the present study.

We used an existing dataset of systematic reviews from a previous project (CRD42019134904) which means that this secondary analysis was not planned when the sample already had been drawn. We share our data including the new IfR variables to ensure transparency and reproducibility of the results in this meta-research project (https://osf.io/y9v4x/).

Analyzing the stop statements as a special type of IfR is a relevant research field. However, stop statement were only reported in three systematic reviews, which does not allow generalization of these findings although they were in line with other meta-research findings [19].

Finally, identifying, classifying, and extracting IfR statements was challenging because of the text-based data structure. Nevertheless, the extraction of IfR variables was thoroughly piloted by three different reviewers. Data extraction was not done in duplicate. However, we had weekly in-depth discussions of arising questions in which consensus was reached. This approach clearly contributed to the validity of the extracted data.

### Implications for research

As a dichotomous approach for IfR data extraction was utilized in this work (e.g., did IfR statement contain the GRADE concept of ‘imprecision’ or related expressions like ‘wide confidence intervals’? yes/no), we suggest that future meta-research studies may elaborate on IfR elements in more detail. For example, concepts underlying GRADE domains could be captured not with a narrow definition (e.g., ‘imprecision’ had to be mentioned literally in IfR statements in our work) but rather assess the concept of imprecision allowing for different expressions like ‘sample size’ or ‘power’ in IfR statements. This would result in nominal IfR variables regarding PICO elements and concepts underlying GRADE domains with more than two categories and would add value for understanding how IfR recommendations are informed. However, we suggest that a distinction should be made in data extraction and communication of IfR between IfR variables describing the shortcomings of the body of evidence (concepts underlying GRADE domains) and IfR recommendations defining a future trial, which are usually described by PICO, study design and additional (methodological) elements, e.g. the suggestion of an IPD meta-analysis.

As a result of the enhanced knowledge of various IfR elements, best practice examples and/or a reporting guideline could be drafted. Best practice examples, e.g. displayed with tables or figures and supporting text, could be evaluated by primary researchers using an online survey. An IfR (reporting) guideline would include all potentially relevant IfR elements and could be further supplemented with suggestions for an appropriate wording based on application examples. Developing such an IfR guideline is an iterative process and should be based on established methods like Delphi study, survey of experts, and iterative group discussions to reach consensus as applied for the development of other well-known reporting guidelines [8, 22–25].

Another field of further research could be the identification of methodological and clinical stop criteria for further studies and the definition of conclusiveness in systematic reviews with or without meta-analysis [19, 20, 26]. This could either be a part of the above-mentioned IfR guideline or result in a separate guideline due to complexity of the question. Patient involvement should play a role in both approaches to ensure that patient values are taken into account when stating IfR [2, 27].

Finally, further meta-research studies could assess if stakeholders like journal editors and/ or peer reviewers explicitly endorse IfR sections. The view and expectations of policy makers, funders, guideline developers and other relevant stakeholders on what IfR sections should include could be captured in (online) surveys.

## Conclusion

About 80% of systematic reviews of our sample included IfR statements. In > 50% of systematic reviews, these IfR statements included ‘intervention’, ‘patient’ and ‘study design’. However, IfR were generally unstructured and incomplete regarding combinations of PICO elements and study design. Concepts underlying GRADE domains were reported rarely in IfR statements.

Additional research is required to determine the generalizability of these IfR results beyond systematic reviews in the context of advanced cancer patients. We recommend the development of more precise guidance on which and how IfR elements to report in systematic reviews of interventions and other types of systematic reviews (e.g., on diagnostic test accuracy). In the interim, one reasonable approach according to the Cochrane Handbook could involve using PICO elements and methodological concepts underlying GRADE to specify IfR.

### Supplementary Information

**Additional file 1.**

## Data Availability

The data that support the findings of this study are available from the Open Science Framework (OSF): https://osf.io/y9v4x/

## Abbreviations

GRADE: Grading of Recommendations, Assessment, Development and Evaluation
IfR: Implications for research
PRISMA: Preferred Reporting Items for Systematic reviews and Meta-Analyses
RCT: Randomized controlled trial
EVBRES: EVidence-Based RESearch
